# Curcumin induces the apoptosis of human monocytic leukemia THP-1 cells via the activation of JNK/ERK Pathways

**DOI:** 10.1186/1472-6882-12-22

**Published:** 2012-03-24

**Authors:** Chu-Wen Yang, Chi-Lun Chang, Hsin-Chen Lee, Chin-Wen Chi, Jia-Ping Pan, Wen-Chin Yang

**Affiliations:** 1 Department of Microbiology, Soochow University, Shih-Lin, Taipei 111, Taiwan, ROC; 2 Institute of Pharmacology, School of Medicine, National Yang-Ming University, Taipei 112, Taiwan, ROC; 3 Department of Medical Research and Education, Taipei Veterans General Hospital, Taipei 112, Taiwan, ROC; 4 Agricultural Biotechnology Research Center, Academia Sinica, Taipei 115, Taiwan, ROC

## Abstract

**Background:**

Curcumin is a principal compound of turmeric, commonly used to treat tumors and other diseases. However, its anti-cancer activity in human acute monocytic leukemia THP-1 cells is not clear. This study aimed to study the anti-cancer effect and action of curcumin on THP-1 cells.

**Methods:**

THP-1 parental cells and PMA-treated THP-1 cells, were used as *in vitro *models to evaluate the anti-cancer effect and mechanism of curcumin. Apoptosis and its mechanism were evaluated by WST-1, flow cytometry and Western blotting. MAPK inhibitors were used to further confirm the molecular mechanism of curcumin-induced THP-1 cell apoptosis.

**Results:**

Curcumin induced cell apoptosis of THP-1 cells as shown by cell viability, cell cycle analysis and caspase activity. Curcumin significantly increased the phosphorylation of ERK, JNK and their downstream molecules (c-Jun and Jun B). Inhibitor of JNK and ERK reduced the pro-apoptotic effect of curcumin on THP-1 cells as evidenced by caspase activity and the activation of ERK/JNK/Jun cascades. On the contrary, the pro-apoptotic effect of curcumin was abolished in the differentiated THP-1 cells mediated by PMA.

**Conclusions:**

This study demonstrates that curcumin can induce the THP-1 cell apoptosis through the activation of JNK/ERK/AP1 pathways. Besides, our data suggest its novel use as an anti-tumor agent in acute monocytic leukemia.

## Background

Acute myeloid leukemia (AML) is a hematopoietic cancer characterized by a disorder in differentiation of hematopoiesis; this disease results in the growth of a clonal population of neoplastic cells. Malignant hematopoietic cells lead to loss of normal hematopoietic functions, which results in death within weeks to months [1]. AML is the most common type of leukemia in adults. It has the lowest survival rate of all leukemia [2]. A better understanding of the molecular biology of AML will be helpful when developing new therapeutic strategies that specifically target molecular abnormalities.

Mitogen-activated protein kinases (MAPKs) such as ERK, JNK and p38 mediate the signaling transduction involved in cell proliferation, differentiation, transformation survival and death [3]. Several publications showed the involvement of MAPKs in the apoptosis of HL-60 cells isolated from the patients with human promyelocytic leukemia, one type of acute myeloid leukemia. For instance, the activation of p38/ERK, JNK/ERK and p38/JNK by anti-cancer compounds, trifolin acetate [4], fucoidan [5] and 3,6-dihydroxyflavone [6], respectively, were observed during HL60 cell death. Accordingly, AP-1 transcription factor is associated with JNK mediated HL-60 cell apoptosis [7-10]. These data support the notion that the MAPKs and the downstream transcription factor AP-1 are the major mediators of HL-60 apoptosis.

Medicinal plants, used in complementary and alternative medicine, are an extraordinary source of chemopreventive and therapeutic agents for various human tumors [11,12]. Turmeric has traditionally been used as a component to treat a variety of disorders in the Indian Ayurvedic medicine. Accumulating evidence shows that curcumin, the principal curcuminoid of turmeric, inhibits proliferation and induce apoptosis in various types of solid tumor and leukemia cell lines [13,14]. Curcumin has been reported to possess inhibitory effects on MDR1 and WT1 gene expression in AML patient leukemic cells [15,16]. Several studies have revealed that curcumin induces HL-60 cell line (a promyelocytic leukemia type of AML) apoptosis through several pathways, including the ornithine decarboxylase-dependent pathway [17], ER stress [18] and an inhibition of telomerase activity [19]. However, little is known about the effects of curcumin on other types of AML.

In the present study, we investigated the effect and mode of action of curcumin on monocytic leukemia THP-1 cells. We first examined the effect of different concentrations of curcumin on THP-1 cell apoptosis. Next, interference of the inhibitor of ERK and JNK and PMA-treated THP-1 cells were used to study the likely mechanism of curcumin-mediated apoptosis.

## Methods

### Cell and reagents

The THP-1 cell line, derived from human acute monocytic leukemia, was purchased from American Type Culture Collection (TIB-202). Cells were cultured in RPMI-1640 (Gibco) supplemented with 10% FBS (Gibco), 10 mM HEPES (GeneMark), 1% L-glutamine (Gibco), 1% non-essential amino acids (Gibco). Curcumin, dimethyl sulfoxide (DMSO), SP600125 (ERK inhibitor), U0126 (JNK inhibitor) and phorbol-12-myristate-13-acetate (PMA) were purchased from Sigma. Antibodies against caspase-3, cleaved caspase-8, Caspase-9, FoxO4, phospho-FoxO4 (Thr28), FoxO3a, FoxO1, phospho-FoxO1 (Ser256), phospho-FoxO3a (Ser253), p85, phospho-p85 (Tyr458), p110α, PDK1, Phospho-PDK1, JunB, c-Jun, phospho-c-Jun Ser63, AKT1, AKT2, AKT3, phospho-AKT (Ser473), phospho-AKT (Ser308), ATF2, phospho-ATF2 Thr71, phospho-JNK (Thr183/Tyr185), phospho-ERK (Thr202/Tyr2040), ERK, JNK, p38, phospho-p38 (Thr180/Tyr182), caspase-8 and histone H3 were purchased from Cell signaling laboratory and antibodies against PARP-1, caspase-3 and GAPDH were from Epitomics Inc. β-actin antibody and phospho-JunB (Ser259) were purchased from Sigma and Santa Cruz Biotechnology, respectively.

### Flow cytometry

THP-1 cells, which had been treated with curcumin (30 μM, 40 μM and 50 μM), were harvested and fixed with 70% ethanol at 4°C overnight. After PBS washing, the cells were incubated with RNase A for 5 min. After incubation with propidium iodide (200 μg/mL), the cells underwent flow cytometry (Beckman, FC-500). For double staining, THP-1 cells were first treated with PhipPhiLux-G1D2/caspase-3 substrate (OncoImmuno, Inc) at 37°C for 45 min. After washing, the cells were stained with propidium iodide and analyzed using flow cytometry.

### Protein extraction and immunoblotting

THP-1 cells were lyzed with RIPA lysis buffer (Sigma). Total cell lysates were extracted as described previously (Chen et al., 2009). The lysates were separated using polyacrylamide gel electrophoresis. After transfer, the membrane was blotted with antibody and developed with an enhanced chemiluminescent kit.

### Caspase activity assay

THP-1 cells were treated with DMSO and curcumin (50 μM) in the presence of U0126 (20 μM) and SP600125 (30 μM) for 10 hours. The cells were subsequently incubated with Caspase-Glo^® ^3/7 reagent kit (Promega) and caspases-3/7 activity was detected and analyzed using a GloMax^®^-Multi Detection System (Promega) according to the manufacturer's instructions.

### WST-1 assays

THP-1 cells and PMA-treated tHP-1 cells were seeded at the density of 50 000 cells/cm^2 ^in 96-well plates. The cells were incubated with DMSO and 50 μM curcumin for 18 hr. After washing, the cells were incubated with WST-1 reagent at 37°C for 1 hr in accordance to the manufacturer's instructions (Roche). The quantity of formazan dye was determined with a photometer at 450 nm.

### Statistics

Data from three independent experiments are presented as mean ± standard deviation (SD). Student's *t*-test was used for statistical analysis between control and treatment groups. *P *less than 0.05 is considered statistically significant.

## Results

### Curcumin induces THP-1 cell apoptosis

**Figure 1:**
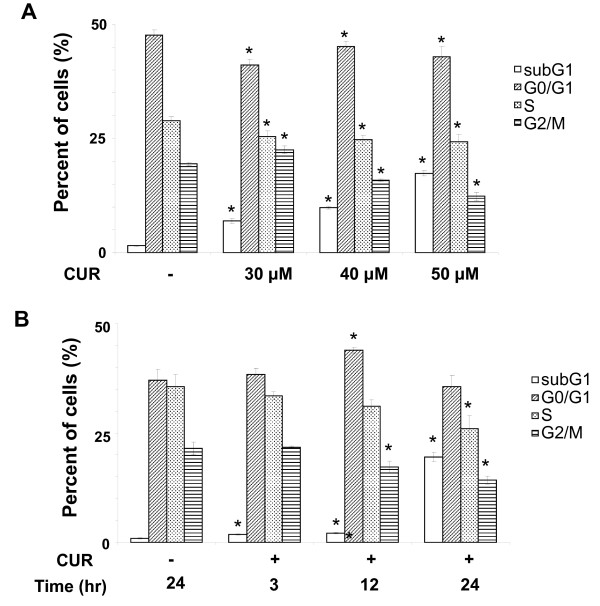
**Effect of curcumin on THP-1 cell apoptosis**. (A) THP-1 cells treated with vehicle (-) and curcumin (CUR, 30, 40 and 50 μM) for 24 hours underwent propidium iodide (PI) staining and flow cytometry. The percentage of the cells in different cell cycle phases was decided and replotted into histogram. Data from three independent experiments are presented as mean ± SD. *P *(*) < 0.05. (B) THP-1 cells treated with vehicle (-) and 50 μM curcumin (CUR) for 3, 12 and 24 hours were subjected to the same procedure as (A). Data from three independent experiments are presented as mean ± SD. *P *(*) < 0.05.

**Figure 2:**
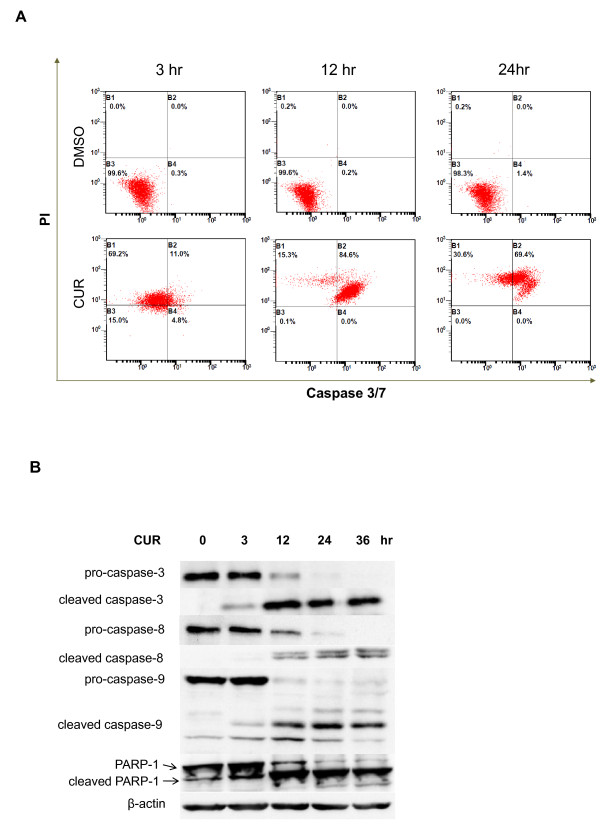
**Effect of curcumin on the activation of caspases in THP-1 cells**. (A) THP-1 cells were treated with vehicle (DMSO) and 50 μM curcumin (CUR) for 3, 12 and 24 hours. The cells were stained with propidium iodide (PI) staining and fluorescent caspase-3/7 substrate. PI intensity and caspase-3/7 activity were analyzed by flow cytometry. The data are representative of 3 experiments. (B) THP-1 cells were treated with 50 μM curcumin (CUR) for 0, 3, 12, 24 and 36 hours. Total lysates of the cells were subjected to SDS-PAGE electrophoresis and blotted with the antibodies against caspase-8, caspase-9, caspase-3 and PARP1. The data are representative of 3 experiments.

To investigate the anti-cancer effect of curcumin on THP-1 cells, a cell line of human monocytic leukemia, THP-1 cells at exponentially growing stage were incubated with different concentrations (30, 40 and 50 μM) of curcumin for 24 hours. DMSO did not affect cell cycle in THP-1 cells (Figure 1A). The subG1 fractions of curcumin-treated THP-1 cells were significantly increased in a concentration-dependent manner (Figure 1A). In contrast, the G2/M fractions were decreased (Figure 1A). However, the G0/G1 and S fractions seemed not to change (Figure 1A). The data suggest that curcumin can induce cell death of THP-1 cells. Furthermore, we studied the time course of cell death of THP-1 cells treated with curcumin. We found that DMSO did not induce THP-1 cell death (Figure 1B). In contrast, curcumin at 50 mM significantly enhanced the subG1 fractions and this enhancement peaked at 24 hours (Figure 1B). Besides, we analyzed the apoptosis of curcumin-treated THP-1 cells using caspase-3/7 activity and propidium iodide staining. The data revealed that curcumin induced THP-1 cell death via apoptotic pathway (Figure 2A). To further study if curcumin activated intrinsic and extrinsic pathways during apoptosis, we examined the cleavage of caspase-8, a caspase in the extrinsic pathway, caspase-9, a caspase in the intrinsic pathway, caspase-3 and PARP-1, substrates of caspases. The results showed the activation of caspases by curcumin started at 3 hours post-treatment, followed by the degradation of PARP-1 (Figure 2B). Taken, together, the data suggest that curcumin concentration-dependently induces THP-1 cell apoptosis through both the extrinsic and intrinsic apoptotic pathways.

### Apoptosis of THP-1 cells by curcumin is not mediated by PI3K/AKT pathway

**Figure 3:**
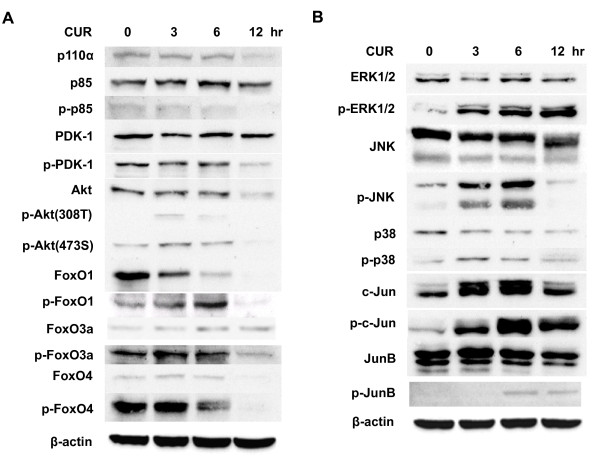
**Effect of curcumin on PI3K/AKT and MAPK pathways in THP-1 cells**. THP-1 cells were treated with 50 μM curcumin (CUR) for 0, 3, 6 and 12 hours. Total lysates of the cells were subjected to SDS-PAGE electrophoresis and blotted with the antibodies against the molecules of PI3K (A) and MAPK (B) pathways. The data are representative of 3 experiments.

PI3K/AKT/FOXO pathway is well known for regulation of cell survival and apoptosis (Burgering and Medema, 2003). Therefore, we examined the involvement of PI3K/AKT/FOXO pathway in the curcumin-mediated apoptosis in THP-1 cells. Figure 3A showed that curcumin treatment did not alter the phosphorylation level of PI3K, AKTs and FOXOs in THP-1 cells.

### Apoptosis of THP-1 cells by curcumin is mediated by the activation of JNK/ERK/Jun pathways

We turned to examine the involvement of MAPK pathways in the curcumin-mediated apoptosis in THP-1 cells. We found that curcumin increased the phosphorylation level of JNK and ERK to a greater extent than p38 in THP-1 cells (Figure 3B). Accordingly, curcumin augmented the phosphorylation of c-Jun and JunB, the downstream transcription factors of JNK and ERK, in THP-1 cells (Figure 3B).

**Figure 4:**
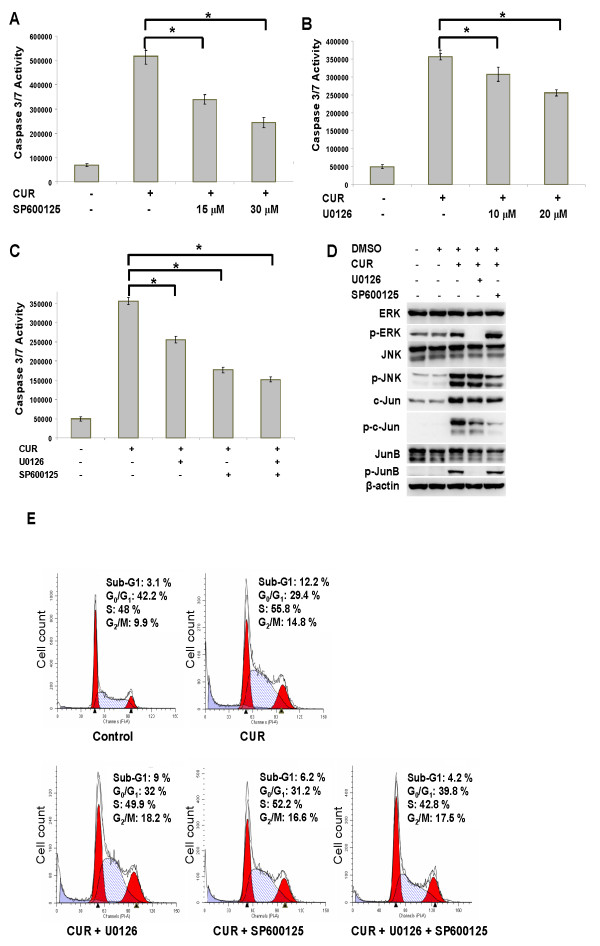
**JNK and ERK inhibitors reduce the apoptotic effect of curcumin in THP-1 cells**. (A) THP-1 cells, which were pre-treated with 50 μM curcumin (CUR) for 2 hours, were incubated with 15 and 30 μM JNK inhibitor (SP600125) for an additional 10 hours. The caspase-3/7 activity of THP-1 cells (1.2 × 10^4^) was determined. Data from 3 independent experiments are presented as mean ± SD. *P *(*) < 0.05. (B) The same procedure as (A) except that the curcumin-treated cells were incubated with 10 and 20 μM ERK inhibitor (U0126). Data from three independent experiments are presented as mean ± SD. *P *(*) < 0.05. (C) The same procedure as (A) except that the curcumin-treated cells were incubated with 20 μM ERK inhibitor (CUR + U0126), 30 μM JNK inhibitor (CUR + SP600125) and both (CUR + SP + U). Data from three independent experiments are presented as mean ± SD. *P *(*) < 0.05. (D) THP-1 cells, which were treated with 50 μM curcumin (CUR) for 2 hours, were incubated with 20 μM ERK inhibitor (U0126) and 30 μM JNK inhibitor (SP600125) for additional 10 hours. Total lysates of the cells were separated and blotted with the indicated antibodies. The data are representative of 3 experiments. (E) THP-1 cells, which were treated with 50 μM curcumin (CUR) for 2 hours, were incubated with 20 μM ERK inhibitor (U0126),30 μM JNK inhibitor (SP600125) or both for additional 10 hours. The cells were stained with propidine iodine (PI), followed by flow cytometry analysis.

To further verify the role of the JNK and ERK pathways in the curcumin-induced THP-1 cell apoptosis, we tested if the inhibitors of JNK (SP600125) and ERK (U0126) could reverse curcumin-mediated apoptosis in THP-1 cells as evidenced by the activity of caspases-3/7. As expected, both inhibitors reduced curcumin-induced caspase-3/7 activity in THP-1 cells in a concentration-dependent manner (Figure 4A and 4B). However, no synergistic or additive effect was observed when these two inhibitors were combined (Figure 4C), implying that JNK and ERK might be redundant in this system. Consistently, Inhibition of ERK reduced the phosphorylation of ERK, JunB and, to a lesser extent, c-Jun (Figure 4D). In sharp contrast, Inhibition of JNK reduced the phosphorylation of JNK and c-Jun (Figure 4D). Besides, the percentage of sub-G1 population in THP-1 cells treated with vehicle and curcumin with/without the inhibitors of ERK, JNK or both was assessed using DNA content assays. Curcumin significantly increased the percentage of the sub-G1 population (i.e., apoptotic cells) of THP-1 cells. This sub-G1 population induced by curcumin was further reduced by the inhibitor of ERK and JNK. A more pronounced reduction in sub-G1 population was observed in THP-1 cells treated with combinational inhibitors (Figure 4E). The data on the reduction of curcumin-mediated THP-1 cell apoptosis by the MAPK inhibitors using DNA content assays is consistent with those obtained from capase-3/7 assays (Figure 4A to 4C). Overall, the data suggest that curcumin modulates apoptosis in THP-1 cells via the activation of JNK/ERK/Jun pathways. ERK and JNK pathways may be parallel and redundant in the curcumin-induced THP-1 cell apoptosis.

### PMA treatment reduces curcumin-induced THP-1 cell apoptosis by inhibiting ERK/JNK/Jun pathways

PMA is known to induce differentiation of THP-1 monocytic cells into macrophage-like cells (Auwerx et al., 1992). Next, we compared the effect of curcumin on PMA-treated THP-1 cells, differentiated/mature monocytic cells, and THP-1 cells using WST-1 assays. We found that cell viability of PMA-treated THP-1 cells and THP-1 cells after curcumin treatment was 25 ± 0.5% and 96 ± 3.7%, respectively. The data suggest that PMA treatment dramatically reversed curcumin-induced THP1 cell death.

**Figure 5:**
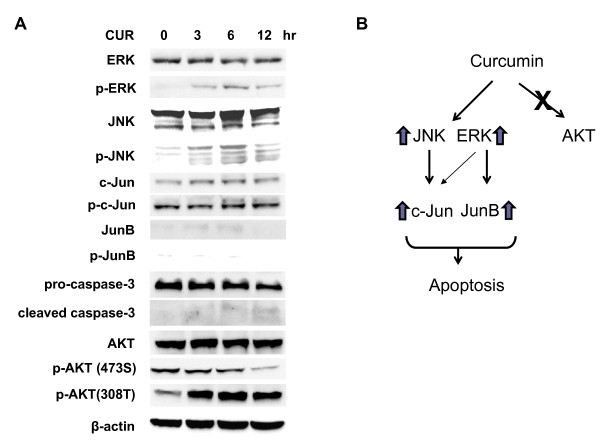
**PMA treatment reverses the effect of curcumin on THP-1 cells**. (A) THP-1 cells were cultured in RPMI-1640 medium with 100 ng/ml PMA for 3 days. The cells were incubated with 50 μM curcumin (CUR) for 0, 3, 6 and 12 hours. After cell lysis, otal lysates of the cells were separated and blotted with the indicated antibodies. The data are representative of 3 experiments. (B) A schematic model describing the mechanism by which curcumin can induce THP-1 apoptosis via MAPK/AP1 regulation.

Next, we examined the effect of curcumin on the ERK/JNK/Jun, caspase-3 and AKT pathways in PMA-treated THP-1 cells. We found that curcumin decreased the phosphorylation of ERK, JNK, c-Jun and JunB and the degradation of caspase-3 in PMA-treated THP-1 cells (Figure 5A) as opposed to THP-1 cells (Figure 3B). In contrast, curcumin increased the phosphorylation of AKT (Figure 5A). The data showed that PMA treatment reversed the apoptotic effect of curcumin on THP-1 cells via the inactivation of ERK/JNK/Jun pathways and activation of AKT pathway. Overall, our data support the notion that curcumin induces the apoptosis of human monocytic leukemia THP-1 cells via the activation of JNK/ERK/Jun pathways (Figure 5B).

## Discussion

In this work, we showed that curcumin induced the apoptosis of THP-1 cells, a human acute monocytic leukemia cell line. This cell death was associated with the MAKP and AP1 pathways. The study proves the concept that curcumin is therapeutically effective against human acute monocytic leukemia, one key type of acute myeloid leukemia.

PI3K, AKT and MAPKs are involved in regulation of life and death [3,20]. Our results showed that curcumin increased apoptosis via the activation of ERK and JNK but not PI3K and AKT (Figure 3). Several anti-cancer compounds such as trifolin acetate [4], fucoidan [5] and 3,6-dihydroxyflavone [6] activated ERK, JNK and/or p38, in human acute myeloid leukemia HL60 cells. The activation of MAPKs was related to apoptosis of HL60 cells [7-10]. Hence, our and other data suggest that MAPKs regulates the matter of life and death in leukemia cells.

Jun family proteins have dual roles in neoplasia and tumor suppression and their roles need to be considered in a context-dependent manner [21-23]. For example, JunB was shown to repress cell proliferation when c-Jun:JunB heterodimers were formed [24,25]. JunB was also reported to have tumor suppressor function in chronic myeloid leukemia [26,27] and B cells [28]. More recently, JunB was shown to inhibit autophagy and induce apoptosis [29,30]. Consistently, AP-1 was shown to be implicated in HL-60 cell apoptosis mediated by JNK [7-10]. In this study, our results showed that c-Jun and JunB are involved in the curcumin-induced apoptosis in THP1 cells (Figures 3B, 4D and 5), suggesting the tumor suppressor role of c-Jun and JunB in THP-1 cells.

Collectively, this study showed that curcumin induces THP-1 apoptosis via the activation of ERK/JNK pathways and its downstream mediators, c-Jun and JunB. The data are in good agreement with the publications indicating that MAPK/AP1 pathways regulated cell death in acute myeloid leukemia HL60 cells. Moreover, our and other data support the notion that the MAPKs and the downstream molecule, AP-1, are the major mediators that regulate cell death of AML tumors.

Leukemic cells are aberrant immature blood cells. Differentiation of leukemic cells is thought as an anti-leukemia approach. PMA, a PKC activator, is known to promote the differentiation of immature THP-1 monocytic cells to mature THP-1 macrophages. Interestingly, the apoptotic effect of curcumin was abolished in PMA-treated THP-1 cells. Surprisingly, phosphorylation of ERK, JNK and Jun by curcumin decreased in PMA-treated THP-1 cells (Figure 5A). Phosphorylation of AKT seemed to increase (Figure 5A). The data suggest that apoptotic effect of curcumin is more effective against immature leukemic cells than mature cells. Put together, curcumin induces human monocytic leukemic THP-1 cell apoptosis via the activation of MAPK/AP1 pathways.

## Conclusions

This work shows the pro-apoptotic effect and mechanism of curcumin in THP-1 cells. Its apoptotic action involves the activation of JNK/ERK/AP1 pathways. Besides, our data imply the novel use of curcumin as anti-leukemia agent.

## Competing interests

The authors declare that they have no competing interests.

## Authors' contributions

CWY conceived the study. CWY and CLC carried out the experiments. JPP helped CLC and CWY in carrying out the experiments. HCL, CWC and WCY participated in designing the experiments. WCY wrote the manuscript. All authors read and approved the final manuscript.

## Pre-publication history

The pre-publication history for this paper can be accessed here:

http://www.biomedcentral.com/1472-6882/12/22/prepub
